# Overview of the Mechanisms that May Contribute to the Non-Redundant Activities of Interferon-Inducible CXC Chemokine Receptor 3 Ligands

**DOI:** 10.3389/fimmu.2017.01970

**Published:** 2018-01-15

**Authors:** Mieke Metzemaekers, Vincent Vanheule, Rik Janssens, Sofie Struyf, Paul Proost

**Affiliations:** ^1^Laboratory of Molecular Immunology, Department of Microbiology and Immunology, Rega Institute, KU Leuven, Leuven, Belgium

**Keywords:** chemokine, CXCR3, G protein-coupled receptor, interferon-γ, leukocyte migration, glycosaminoglycan, inflammation, posttranslational modification

## Abstract

The inflammatory chemokines CXCL9, CXCL10, and CXCL11 are predominantly induced by interferon (IFN)-γ and share an exclusive chemokine receptor named CXC chemokine receptor 3 (CXCR3). With a prototype function of directing temporal and spatial migration of activated T cells and natural killer cells, and inhibitory effects on angiogenesis, these CXCR3 ligands have been implicated in infection, acute inflammation, autoinflammation and autoimmunity, as well as in cancer. Intense former research efforts led to recent and ongoing clinical trials using CXCR3 and CXCR3 ligand targeting molecules. Scientific evidence has claimed mutual redundancy, ligand dominance, collaboration or even antagonism, depending on the (patho)physiological context. Most research on their *in vivo* activity, however, illustrates that CXCL9, CXCL10, and CXCL11 each contribute to the activation and trafficking of CXCR3 expressing cells in a non-redundant manner. When looking into detail, one can unravel a multistep machinery behind final CXCR3 ligand functions. Not only can specific cell types secrete individual CXCR3 interacting chemokines in response to certain stimuli, but also the receptor and glycosaminoglycan interactions, major associated intracellular pathways and susceptibility to processing by particular enzymes, among others, seem ligand-specific. Here, we overview major aspects of the molecular properties and regulatory mechanisms of IFN-induced CXCR3 ligands, and propose that their *in vivo* non-redundancy is a reflection of the unprecedented degree of versatility that seems inherent to the IFN-related CXCR3 chemokine system.

## General Introduction to the Chemokine Network

*Chemo*tactic cyto*kines* or chemokines are low molecular mass proteins (± 8–12 kDa) with a hallmark function of directing leukocyte migration in a time- and site-dependent manner (1–6). Obviously, controlled chemotaxis of specific leukocyte subtypes is essential not only in homeostatic processes including immune cell homing, embryogenesis, and angiogenesis, but also in pathophysiological environments such as cancer, inflammation and autoimmunity (7–12). As such, chemokines are key players in innate and adaptive immune events, during health and disease. The conventional receptors through which they exert their biological functions are specific G protein-coupled receptors (GPCRs) that mainly activate the inhibitory type of G alpha (Gα_i_) proteins, subsequently eliciting inhibition of adenylate cyclase, thereby reducing concentrations of intracellular cyclic adenosine monophosphate ([cAMP]_i_) (2, 10). However, also G protein-independent signaling may be activated, among which β-arrestin-associated pathways are probably most intensely studied (13). In addition to interaction with specific GPCRs, chemokine availability, activity and receptor preference is modulated at multiple levels including chemokine interactions with glycosaminoglycans (GAGs), atypical chemokine receptors (ACKRs), gene transcription, mRNA stability, alternative gene splicing, mutual synergism or antagonism, and posttranslational modifications (14–17). Thus, the final chemokine functioning *in vivo* is the complex outcome of numerous regulatory mechanisms, emphasizing that an apparently important degree of specificity rather than redundancy may be inherent to the chemokine system.

**Figure 1:**
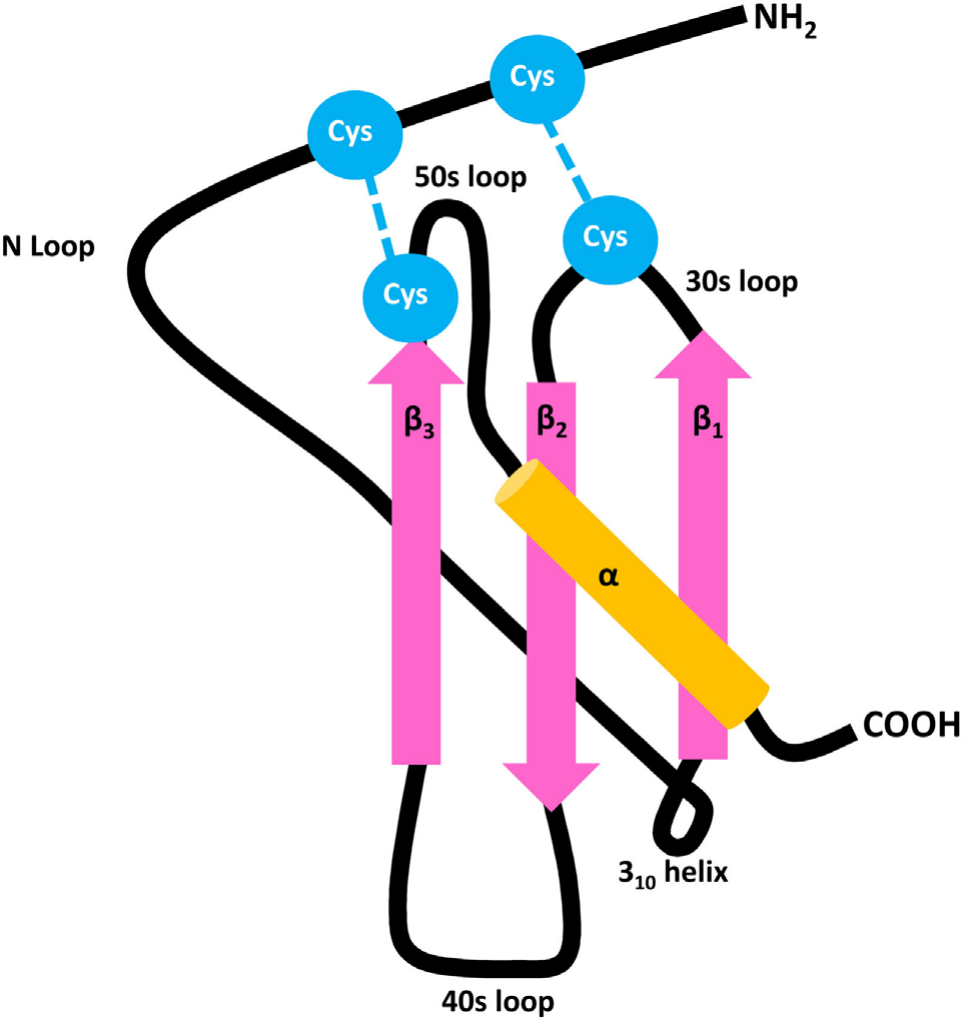
General structure of CXC chemokines. Chemokines contain three antiparallel β strands (pink) and a COOH-terminal α-helix (orange), mutually connected by 30s, 40s, and 50s loops. The flexible NH_2_-terminal domain is followed by an N loop and 3_10_ helix, respectively. The 3D structure of the mature secreted protein is stabilized by two disulfide bridges which are formed by four conserved Cys residues (blue).

With respect to major biological functions, it was originally proposed that the chemokine family can be subdivided into homeostatic and inflammatory proteins that are, respectively, constitutively expressed or require prior induction by endogenous (e.g., cytokines) or exogenous (e.g., microbial products) stimuli (18–21). However, meanwhile it became clear that this subdivision is non-absolute since many chemokines, such as CXCL12, serve both homeostatic and inflammatory roles. Based on the number and positioning of conserved Cys residues present in the NH_2_-terminal sequence of the mature secreted protein, chemokines are structurally classified as CXC, CC, C, or CX3C ligands (5, 10, 22). CC chemokines contain two adjacent NH_2_-terminal Cys and form one of the two largest chemokine subfamilies. The other major subfamily is constituted by CXC chemokines that contain one random (“X”) amino acid in between their NH_2_-terminal Cys residues (Figure 1). Classification of chemokine receptors is complementary to their predominantly recognized chemokine subfamily, with CC chemokine receptors (CCRs) binding CC chemokines, CXC chemokine receptors (CXCRs) interacting with CXC chemokines, *etcetera* (10). A specific chemokine may recognize one or multiple receptors of its complementary subclass, and *vice versa*, thereby conferring an outstanding promiscuity to the chemokine network. To add even more complexity, over the past few years it has been evidenced that a chemokine receptor may preferentially activate one out of several intracellular signaling pathways (13). This phenomenon is known as biased signaling and likely depends not only on the receptor and ligand involved, but also on the cell type or tissue studied.

**Figure 2:**
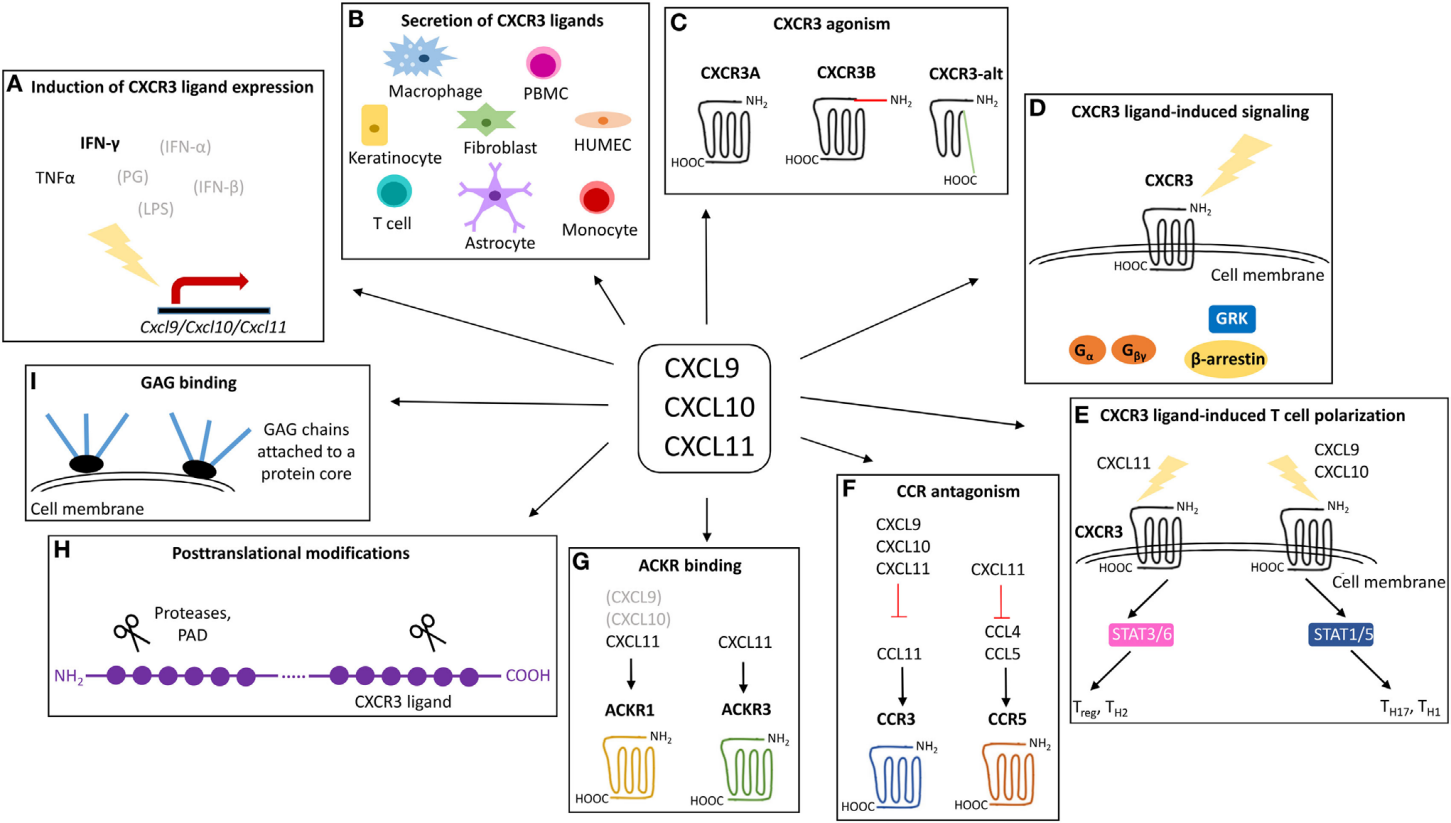
Overview of the mechanisms that may contribute to the exclusivity of CXCR3 ligands. CXCL9, CXCL10, and CXCL11 are structurally related chemokines that share CXCR3 as common receptor and IFN-γ as predominant inducer. Despite structural and functional similarities, emerging evidence points toward non-redundant roles for CXCL9, CXCL10, and CXCL11 *in vivo*. The exclusivity of individual IFN-inducible CXCR3 ligands may be rooted at multiple levels including secretion of specific IFN-inducible CXCR3 ligands by specific cell types in response to specific inducers **(A,B)**, specific CXCR3 interaction features and the existence of different CXCR3 isoforms **(C)**, major associated signaling cascades **(D)**, effects on T cell polarization **(E)**, CCR antagonism **(F)**, ACKR interactions **(G)**, posttranslational processing **(H)**, and GAG binding characteristics **(I)**. ACKR, atypical chemokine receptor; CCR, CC chemokine receptor; CXCR, CXC chemokine receptor; GAG, glycosaminoglycan; GRK, G protein-coupled receptor kinases; HUMEC, human microvascular endothelial cell; IFN, interferon; LPS, lipopolysaccharide; PAD, peptidylarginine deiminase; PBMC, peripheral blood mononuclear cell; PG, peptidoglycan; STAT, signal transducer and activator; TNF, tumor necrosis factor.

Seven of the human CXC chemokines, i.e., CXCL1–3 and CXCL5–8, contain a conserved Glu-Leu-Arg (“ELR”) amino acid motif (5, 10, 22). Human CXCL6 and CXCL8 signal through CXCR1 and all seven ELR^+^ CXC chemokines activate CXCR2 and activation of these receptors results in neutrophil chemotaxis. In addition, CXCR2 ligands and CXCL12, the unique ligand for CXCR4, have been reported to stimulate angiogenesis (9, 23). Most CXC chemokines that lack the ELR motif interact with CXCR3 (5). Regarding these CXCR3 ligands, one may discriminate between platelet-related agonists CXCL4 and CXCL4L1 on the one hand, and CXCL9, CXCL10, and CXCL11 that share interferon (IFN)-γ as a major inducer, on the other hand (24). Although they share a unique receptor and major inducer, emerging evidence points toward non-redundant roles for the three IFN-induced CXCR3 ligands *in vivo* (25). Specifically, it was proposed that, during the course of immune responses, differential stimuli induce CXCL9, CXCL10, and CXCL11 expression by specific cell types, contributing to unique temporal and spatial expression of IFN-inducible CXCR3 ligands. Additionally, their non-redundant biological roles *in vivo* are probably a consequence of multidimensional regulation of the specific activity of IFN-induced CXCR3 agonists as indicated by, for example, ligand-specific receptor- and GAG-binding features, major associated intracellular signaling pathways and differential susceptibility to enzymatic processing. In the present review, we overview the IFN-inducible CXCR3 chemokine system and focus on aspects that may contribute to the non-redundant activities of individual IFN-induced CXCR3 chemokines *in vivo* (Figure 2).

## Discovery and Expression of IFN-Inducible CXCR3 Chemokines

In 1985, a study aiming to detangle the IFN-γ-mediated inflammatory response noticed a gene encoding a protein with high homology to platelet-derived proteins (26). The molecular mass of the protein was approximately 10 kDa and it was named “IFN-γ-inducible protein of 10 kDa” (IP-10). Five years later, in 1990, an mRNA encoding another platelet factor-4-like protein selectively induced by IFN-γ and no other macrophage activators, including IFN-α, IFN-β, and lipopolysaccharide (LPS), was described (27). The authors proposed that the molecule should be named “monokine induced by IFN-γ” (Mig). It became clear that IP-10 and Mig were highly similar proteins, with their corresponding genes located on the q21.1 region of chromosome 4 in a head-to-tail orientation, and their start codons separated by not more than 16 kb (28). Ensuing studies revealed that IP-10 and Mig are *chemo*tactic cyto*kines* or chemokines that lack a conserved ELR amino acid motif and contain two conserved Cys residues separated by one random residue (“X”) in their NH_2_-terminal sequences. They both act on CXCR3, which was originally reported as a selective receptor for these two chemokines (29). Subsequently, two research groups identified a third ELR negative, IFN-inducible CXC chemokine in stimulated astrocytes and keratinocytes (30, 31). This protein was strongly related to IP-10 and Mig and displayed an even higher affinity for CXCR3. This third IFN-associated CXCR3 ligand was named “IFNγ-inducible protein-9 (IP-9)” or “IFN-inducible T cell α chemoattractant” (I-TAC) in the first publications and the corresponding gene was found in the same 4q21.1 chromosomal mini cluster (30, 32). In the now established systematic chemokine nomenclature Mig, IP-10, and I-TAC/IP-9 were renamed CXCL9, CXCL10, and CXCL11, respectively (22), and are commonly referred to as IFN-inducible CXCR3 ligands.

The IFN-inducible CXCR3 chemokines show circa 40% homology in their amino acid sequences and are produced by a variety of cells including human microvascular endothelial cells (HUMEC), keratinocytes and fibroblasts (Table 1; Figure 2) (30, 31, 33–35). Additionally, CXCL9 and CXCL11 are commonly secreted by peripheral blood mononuclear cells (PBMCs) and more specifically by macrophages (CXCL9) (27) and astrocytes (CXCL11) (30). Leukocytes that predominantly produce CXCL10 are T cells, and monocytes (34, 36, 37). In addition to differential major cellular origins, unique promotors control the expression of individual IFN-inducible CXCR3 interacting chemokines (Figure 2). The *Cxcl9* promotor holds a γ-interferon response element (γIRE) and a nuclear factor kappa B2 (NF-κB2) site, and CXCL9 protein expression truly depends on IFN-γ (38–40). The *Cxcl10* and *Cxcl11* promotors show a certain degree of similarity as they both are induced by IFN-γ, contain an interferon response element (IRSE) and an NF-κB1 site (38, 41–43). The IRSE in the *Cxcl10* promotor mediates responsivity of the gene to IFN-α and IFN-β. Thus, both Type I and Type II IFNs are potent inducers of CXCL10 expression. Moreover, various innate stimuli recognized by innate immune sensors can induce IFN-α production by immune cells and therefore may indirectly promote CXCL10 production (44). Remarkably, CXCL11 is induced by IFN-β and IFN-γ, but not by IFN-α (45). Additionally, tumor necrosis factor (TNF)-α alone weakly induces CXCL10. For the three IFN-inducible CXCR3 ligands, gene transcription induced by the respective IFNs is strongly enhanced in the presence of TNF-α and IL-1β in fibroblasts and endothelial cells (46). Surprisingly, although bacterial LPS and peptidoglycans also synergistically induced the three CXCR3 ligands in fibroblasts and endothelial cells, they inhibited IFN-induced production of the CXCR3 ligands by leukocytes (33–35). In addition, at a single cell level, endothelial cells were clearly better producers of the CXCR3 ligands than fibroblasts and leukocytes (33–35).

**Table 1:** Major sources of natural IFN-inducible CXCR3 ligands.

Cell type	Species	Stimulation	Produced CXCR3 ligand	Reference
Macrophage cell line RAW264	Murine	IFN-γ	CXCL9	(27)

Astrocytes	Human	IFN-γ + TNF-α + IL-1β	CXCL11	(30)

Keratinocytes	Human	IFN-γ	CXCL10, CXCL11	(31, 37)
		Purified protein derivative of tuberculin	CXCL10	(37)

Endothelial cells	Human	IFN-γ alone or synergistically with LPS, PG or dsRNA	CXCL9, CXCL10	(33, 37)
		IFN-γ alone or synergistically with dsRNA	CXCL11	(33)
		Purified protein derivative of tuberculin	CXCL10	(37)
		IL-1β or TNF-α plus IFN-α, IFN-β, or IFN-γ	CXCL10	(46)

Fibroblasts	Human	Purified protein derivative of tuberculin, IFN-γ alone or synergistically with LPS, PG or dsRNA	CXCL9, CXCL10, CXCL11	(35, 37)
		IL-1β or TNF-α plus IFN-α, IFN-β, or IFN-γ	CXCL10	(46)

PBMCs	Human	IFN-γ or dsRNA, inhibited by PG	CXCL9, CXCL11	(35, 47)

Monocytes	Human	IFN-γ or dsRNA, inhibited by PG	CXCL10	(34, 48)

T cells	Human	PHA with or without PMA	CXCL10	(36)

Dermal macrophages	Human	Purified protein derivative of tuberculin, IFN-γ	CXCL10	(37)

*dsRNA; double-stranded RNA; IFN, interferon; LPS, lipopolysaccharide; PBMCs, peripheral blood mononuclear cells; PG, peptidoglycan; PMA, phorbol 12-myristate 13-acetate; PHA, phytohemagglutinin; TNF-, tumor necrosis factor*.

## CXCR3

### Identification and Expression of CXCR3

**Figure 3:**
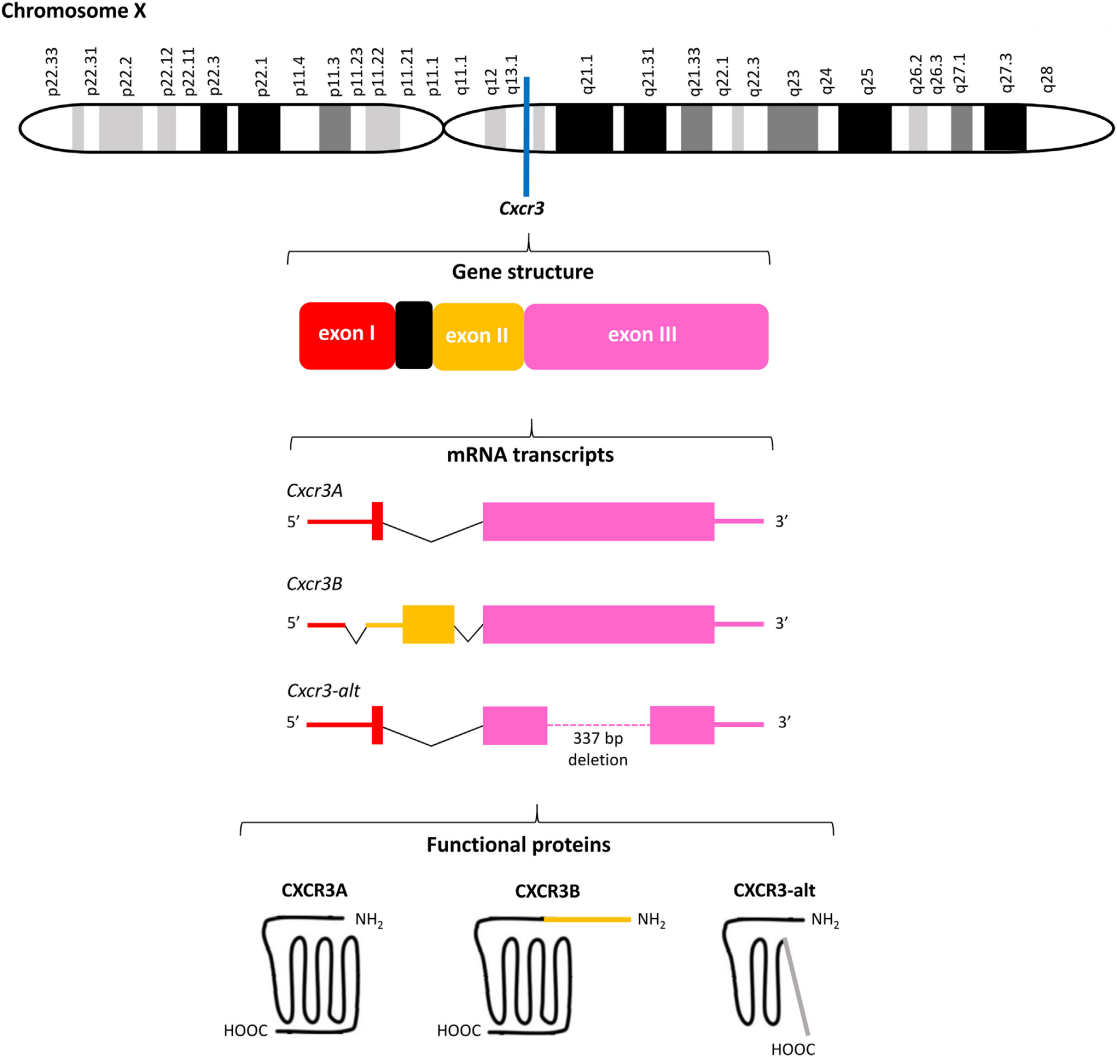
Overview of the *Cxcr3* gene structure. *Cxcr3* is located at the q13.1 region on chromosome X and contains three exons and one intron. Alternative splicing generates three mRNAs encoding three structurally and functionally different CXC chemokine receptor 3 (CXCR3) proteins. The canonical CXCR3A contains 368 amino acids. The four most NH_2_-terminal residues are encoded by exon I of *Cxcr3* and all remaining amino acids are encoded by exon III. CXCR3B (415 amino acids) contains a unique NH_2_-terminal tail of 51 amino acids encoded by exon II. Both CXCR3A and CXCR3B contain seven transmembrane domains. The significantly shortened CXCR3-alt (267 amino acids) results from posttranscriptional exon skipping and contains only four or five transmembrane domains.

The human chemokine receptor CXCR3 was described for the first time in 1996 (29). The receptor was originally labeled “the first lymphocyte chemokine receptor that was not coexpressed by monocytes or granulocytes” (29). The corresponding gene was found two years later and, strikingly, is located on chromosome X, at the q13.1 region (Figure 3) (49). The gene encodes a multi-pass membrane molecule of 368 amino acids with a molecular mass of nearly 41 kDa (29). CXCR3 is a class A GPCR encompassing seven transmembrane helices. The receptor is predominantly expressed on activated T cells. Meanwhile, CXCR3 has been detected on regulatory T cells, CD4 positive and CD8 effector and memory T cells, with higher levels detected on T helper (Th)1 cells compared to Th2 cells (29, 49–62). Dendritic cell (DC)-mediated T cell activation efficiently induces CXCR3 on naive T lymphocytes, which are initially CXCR3 negative. Also in cell cultures, interleukin (IL)-2 with or without phytohemagglutinin (PHA) can upregulate CXCR3 on naive cells with high efficiency, resulting in approximately 95% CXCR3 positivity of the total culture (49). Other subtypes of leukocytes, e.g., innate lymphoid cells (ILCs), γδT cells, natural killer (NK) cells, NKT cells, specific B lymphocytes and DCs themselves, may also express functional CXCR3 (63–68). Furthermore, expression of CXCR3 was evidenced on various cells that are not related to the immune system. These include fibroblasts, endothelial and epithelial cells, but also astrocytes and smooth muscle cells (63, 69). More recently, CXCR3 was found on eosinophils and neutrophils in an inflamed environment (70–72). Thus, implying that the dogma stating that CXCR3 is not present on granulocytes requires adjustment.

### Discovery of CXCR3 Variants Generated by Alternative Splicing

Meanwhile, the originally described CXCR3 protein of 368 amino acids has been renamed CXCR3A and two other CXCR3 isoforms, resulting from alternative splicing of the *Cxcr3* gene, were discovered (Figure 3). Several studies claimed that unique downstream signaling cascades, functions and expression patterns can be attributed to individual CXCR3 variants (73, 74). Indeed, evidence exists that independent on the presence of ligands, CXCR3 variants may be differentially expressed in specific cell types and activate partially different signal transduction pathways, suggesting that alternative gene splicing may play a role in fine-tuning the context-specific role of CXCR3 and its ligands *in vivo* (23, 73–76). CXCR3A, the most abundant form, interacts with CXCL9, CXCL10, and CXCL11 to induce chemotaxis and calcium mobilization. CXCL11 and CXCL10 induce activation of the inhibitory type of Gα proteins (Gα_i_), β arrestin-1 and β arrestin-2 recruitment, and ERK1/2 phosphorylation (74, 77). Although the response upon treatment of CXCR3A with CXCL9 is in general weaker than with CXCL10 and CXCL11, in HEK293T cell transfectants all three ligands efficiently induced receptor internalization (74). Coupling to Gα_i_ proteins implies that activation of CXCR3A provokes inhibition of adenylyl cyclase activity and a subsequent decrease of endogenous [cAMP]_i_ concentrations. This downstream signaling ultimately elicits an increase of the intracellular calcium concentrations ([Ca^2+^]_i_), cell proliferation and initiation of migration-related cellular responses (29, 49). Alternative splicing at the 5′ end of exon 2 of *Cxcr3* generates the less commonly expressed CXCR3B of 415 amino acids. This second CXCR3 variant contains a unique NH_2_-terminal tail of 51 amino acids that replaces the four most NH_2_-terminal residues of CXCR3A. At the mRNA level, CXCR3A and CXCR3B were found in heart, kidney, liver, and skeletal muscle tissues, while CXCR3A was also present in placenta (73). Although immune cells mainly express CXCR3A, they usually coexpress low levels of CXCR3B (24). Moreover, endothelial cells may selectively express CXCR3B. The two platelet-derived chemokines CXCL4 and CXCL4L1, in addition to the three IFN-inducible CXCR3 chemokine ligands, also bind to CXCR3A and CXCR3B (73, 78). Lastly, CXCR3-alt results from posttranscriptional exon skipping and contains only four or five transmembrane regions and 267 amino acids (75). This significantly shortened CXCR3 isoform was originally reported to bind solely CXCL11 with low affinity, resulting in a moderate increase of the [Ca^2+^]_i_ and chemotaxis (75, 76).

Among the IFN-inducible CXCR3 agonists, CXCL10 exhibits the highest binding affinity for CXCR3B (73). All IFN-inducible CXCR3 ligands display a higher affinity for the canonical CXCR3A than CXCR3B. Moreover, chemokine signaling through CXCR3B is not associated with a calcium flux. Compared to CXCR3A transfectants, p21 mRNA levels were ten times higher in cells transfected with CXCR3B (73). The cyclin-dependent kinase inhibitor p21 has an essential role in linking DNA damage to cell cycle arrest (79, 80) and induction of its expression is proposed to be part of the machinery involved in the antiproliferative response resulting from stimulatory G alpha (Gα_s_) protein signaling and the subsequent increase of the [cAMP]_i_ (81). Consequently, it was suggested that, in contrast to most conventional chemokine receptors and CXCR3A, CXCR3B may couple to Gα_s_ proteins upon ligand-interaction on microvascular endothelial cells, explaining the [cAMP]_i_ increase originally claimed to this CXCR3 variant. Moreover, the authors proposed that these differences in Gα protein coupling explained the contradictory cellular responses induced *via* CXCR3A and CXCR3B (73). Evidence favoring this hypothesis was provided by the observation that CXCR3B activation initiates an antiproliferative response and negatively affects cell migration. Also, CXCR3B was believed to be the receptor responsible for the antiangiogenic effects of CXCR3 ligands (73). However, no Gα_s_ stimulation could be observed in CXCR3B transfected HEK293T cells and in mice no CXCR3B form exists whereas the CXCR3 ligands and in particular CXCL4L1 retains potent antiangiogenic activity in these animals (74, 78, 82).

### Potential Significance of Splice Variants for the *In Vivo* Function of CXCR3

Meanwhile, somewhat controversial results regarding CXCR3B and CXCR3-alt agonists and signaling were published, underscoring the current need to examine the physiological relevance of CXCR3 variants resulting from alternative gene splicing in an *in vivo* context. Furthermore, it cannot be excluded that contrasting results may be obtained in individual studies focusing on CXCR3 variants, depending on the experimental model used. Indeed, a cell-dependent rather than CXCR3 splice variant-dependent mechanism may be decisive for the major subtype of Gα protein (Gα_i_ or Gα_s_) activated upon ligand stimulation. Moreover, this would at least partially explain why also murine CXCR3, which exists in only one isoform and was originally considered a classical Gα_i_-coupled chemokine receptor, mediates angiostatic effects (78). Considering CXCR3/Gα_i_-signaling in mice, interestingly, the critical Gα_i_ protein is Gα_i2_, while Gα_i3_ exerts an inhibitory effect in this animal model (83). The fact that alternative splicing is claimed for human *Cxcr3* but not for the corresponding gene in mice, is one of numerous examples underscoring the potential major differences between the human chemokine network and its murine counterpart.

Due to the limited number of studies with isoform-specific antibodies, the precise contribution of CXCR3 splice variants to the general IFN-related CXCR3 chemokine network in health and disease remains largely unknown. In an inflamed cellular environment, usually a specific chemokine receptor and its ligands are present, and may even be coexpressed by individual cells. This phenomenon adds a second layer of complexity when aiming to detangle the specific *in vivo* contributions of receptor splice variants to CXCR3–chemokine communications. Lasagni et al. reported in the article that originally described CXCR3B that CXCR3A and CXCR3B are both expressed on T cells (73). However, CXCR3B, in contrast to CXCR3A, was expressed on HUMECs and they showed that the CXCR3 ligands inhibit the growth of these microvascular endothelial cells through CXCR3B. In contrast, human mesangial cells primarily expressed CXCR3A and not CXCR3B (73). Differential expression of the three CXCR3 spliced variants was also reported in patients with ovarian carcinoma. Ovarian cancer tissue revealed the highest CXCR3-alt expression (CXCR3-alt expression in cancer > endometriosis > normal tissue) (84). The highest CXCR3B expression was seen in normal tissue (CXCR3B expression in normal > endometriosis > cancer tissue) and CXCR3A was higher in endometriosis and cancer tissue than in normal tissue. Moreover, the CXCR3-alt-high cancer tissue was characterized by low CXCL4 and high CXCL11 expression (84). Also, reduced mRNA levels of canonical CXCR3A, but increased mRNA levels of CXCR3-alt, were found in CD3 positive lymphocytes in peripheral blood from patients with Crohn’s disease (85). Although further research is required, these findings suggest that CXCR3-alt may be a potential biomarker for Crohn’s disease and point toward IFN-inducible CXCR3 ligands (which are strongly produced by the colonic epithelium of these patients) as crucial players in the underlying disease mechanisms. Additionally, enhanced mRNA levels of CXCR3A and decreased mRNA levels of CXCR3B were found in prostate cancer samples (86). Moreover, the authors demonstrated that these altered expression levels were translated into an altered migration and invasion behavior of cancer cells, and proposed that the final outcome of CXCR3A upregulation and CXCR3B downregulation favors tumor progression and metastasis.

## Receptor Interactions of IFN-Inducible CXCR3 Ligands

### General Aspects of CXCR3–Chemokine Interactions

In general, chemokines first bind to their receptors with high affinity, followed by ligand-induced receptor activation. Thus, the chemokine–receptor interaction is considered a two-step mechanism (21, 87, 88). The N loop located in the chemokine core domain plays a crucial role in the initial binding to the NH_2_-terminal domain of the receptor (Figure 1). The subsequent receptor activation is mediated by interaction between the NH_2_-terminal chemokine domain and various receptor regions. Regarding the interaction between CXCR3 and its IFN-inducible agonists CXCL9, CXCL10, and CXCL11, it was found that charged amino acids in the extracellular regions of CXCR3 are important in ligand binding (89). The second extracellular loop, specifically an Arg residue at position 216, is responsible for receptor activation, but not ligand-binding and receptor internalization (90). Sulfation of Tyr27 and Tyr29 residues in its NH_2_-terminal tail is required for CXCR3 activation by the three IFN-inducible ligands. Two additional Tyr residues are present in the NH_2_-terminal extension of CXCR3B at positions 6 and 40. N-glycosylation of CXCR3 expressed on fibroblast-like synoviocytes was confirmed at residues Asn22 and Asn32, with deglycosylation of Asn22 resulting in reduced CXCL10 binding while leaving the CXCR3 expression and stability unaffected (91).

### Ligand-Specific Features of CXCR3–Chemokine Interactions

The receptor interaction modes of individual IFN-inducible CXCR3 agonists also display certain ligand-specific features. Binding of CXCL10 and CXCL11, but not CXCL9, requires the first sixteen NH_2_-terminal residues of CXCR3 (90). CXCL11 possesses the strongest CXCR3-binding affinity, hierarchically followed by CXCL10 and CXCL9 (29, 30). For CXCL10, two hydrophobic clefts, formed by its N loop and 40s region, and by its NH_2_-terminal domain and 30s loop, respectively, were proposed to be the major regions for CXCR3 interaction (Figure 1) (92). Also for CXCL11, the N loop face of the chemokine is critically involved in CXCR3 binding, and displays an even higher degree of hydrophobicity compared to CXCL10 (93). This increased hydrophobicity, but also the observation that the CXCR3 binding face of CXCL11 exhibits more flexible structural elements, may explain its higher affinity for CXCR3. Despite the different receptor affinities of IFN-inducible CXCR3 ligands, mutual competition for CXCR3 binding seems incomplete, and it was therefore suggested that they partially act as allotropic agonists, at least when considering the receptor in its G protein-coupled state (94). In addition, upon uncoupling CXCR3 from G protein-dependent signaling, a phenomenon believed to reflect a situation of relative deficiency of the appropriate G protein, CXCL10/CXCR3 binding is completely abrogated while interaction between CXCL11 and the receptor is only reduced. These observations suggest that these two IFN-inducible CXCR3 ligands bind differential CXCR3 states and may at least partially explain the finding that the maximal saturation value of CXCL11, i.e., maximum amount of chemokine which can bind specifically to CXCR3 in a certain experimental setup, is probably 7–13 times higher compared to CXCL10 (94). Furthermore, their receptor affinities directly correlate with the potencies of the IFN-inducible CXCR3 agonists. Thus, the high-affinity CXCR3 ligand CXCL11 also induces chemotaxis and [Ca^2+^]_i_ mobilization most potently (30). For human CXCR3, chemotaxis and [Ca^2+^]_i_ mobilization induced by all IFN-inducible ligands relies on the COOH-terminal receptor region and conserved DRY motif located in its third transmembrane domain (95). Although the optimal chemokine concentrations for induction of chemotaxis are usually rather low, exposure to high ligand concentrations or prolonged stimulation may result in internalization of the receptor. The three IFN-inducible CXCR3 ligands not only differ in their potency to induce receptor internalization, but also in their mode of action. Whereas CXCL9- or CXCL10-provoked CXCR3 internalization relies on Ser and Thr residues in the COOH-terminal receptor tail and the adaptor protein β-arrestin 1, CXCL11-mediated internalization of the receptor involves its third intracellular loop with no absolute requirement for β-arrestins 1 or 2 (95–97).

### Chemokine-Induced G Protein and β-Arrestin Signaling through Differential CXCR3 Variants

Using HEK 293T cells transfected with individual CXCR3 isoforms, a recent study aimed to provide new insights into the consequences of alternative *Cxcr3* gene splicing at the signaling level (74). However, when comparing receptor isoforms on transfected cells, one has to keep in mind that observed differences may be a result of the technical insufficiency to succeed in equal expression of each isoform instead of being a true biological phenomenon. Remarkably, although CXCL9 induces chemotaxis of CXCR3 positive cells, it is much weaker than CXCL10 and CXCL11 in inducing substantial Gα_i_-activation through CXCR3A. Unlike for CXCR3A, chemokine-induced CXCR3B-signaling was originally associated with a cAMP increase implying that this receptor variant couples to Gα_s_ proteins in microvascular endothelial cells (73, 98). In contrast to these initial findings, it was reported that high concentrations of CXCL11, but no other CXCR3 agonists, may activate Gα_i_-signaling *via* CXCR3B in HEK293T cell transfectants (74). It remains to be elucidated whether cotranslational sulfation of its additional Tyr residues, which are potential sulfation sites, may affect the potency of the CXCR3B variant to signal in a Gα_i_-dependent manner (74). In the same study, no evidence was found for CXCR3B-signaling through Gα_s_ upon stimulation of HEK293T cell transfectants with any of its chemokine ligands. In addition to differential Gα protein interactions, scientific evidence suggests that CXCR3 isoforms also differ regarding their potencies to recruit β-arrestin adaptor proteins. β-arrestins are probably best known for their role in uncoupling GPCRs, including chemokine receptors, from conventional G protein-dependent pathways, thereby promoting receptor desensitization and internalization. Emerging evidence, however, has pointed toward a more complex role for β-arrestins in chemokine-induced signaling (99, 100). Specifically, β-arrestins are believed to function as scaffolding molecules at the leading edge that may fulfill complex roles in modulating numerous intracellular signaling cascades (101–104). For CXCR3, a crucial *in vivo* role for β-arrestin 2 in lymphocyte chemotaxis was demonstrated in mice (105), while multiple studies reported that internalization of CXCR3A involves β-arrestin-independent mechanisms (74, 96, 97, 106). CXCR3A associates with β-arrestins 1 and 2 even without prior chemokine stimulation but CXCR3B preferentially recruits β-arrestin 2 in the absence of chemokines (74). CXCL10 and CXCL11 further enhance recruitment of both β-arrestins to CXCR3A, but stimulation with CXCL9 only slightly increases association between β-arrestin 2 and the receptor. Regarding CXCR3B/β-arrestin interactions, CXCL11 seems to be the only chemokine with a stimulating effect (97, 106). CXCR3-alt was unable to signal through either Gα_i_ or β-arrestins. In addition to differential, ligand-specific potencies of the IFN-inducible CXCR3 agonists to induce β-arrestin recruitment, also β-arrestins’ intracellular redistribution seems to occur in a ligand-dependent manner (106). β-arrestin *puncta* at the membrane were found upon stimulation with CXCL9 and CXCL10, whereas CXCL11 rather induces colocalization of CXCR3 and β-arrestins into endosomes (97, 106). While in the early phase (after a few minutes) CXCR3A-mediated ERK phosphorylation was mediated through G protein-dependent signaling and occurred with all three ligands, CXCR3B-mediated ERK phosphorylation was dependent on β-arrestins (97). In addition, CXCR3A activation also resulted in a late phospho-ERK signal which was not detected upon treatment of CXCR3B transfected cells.

### Chemokine-Induced CXCR3 Internalization

CXC chemokine receptor 3 displays constitutive internalization which can be further enhanced in the presence of its chemokine agonists (96). CXCL11 was found the predominant IFN-inducible CXCR3 ligand responsible for induction of CXCR3 internalization following contact between T cells and stimulated endothelial cells (107). In addition, also in a study using CXCR3 transfected HEK cells, CXCL11 was claimed to be the major chemokine promoting CXCR3 internalization (106). These data could imply that CXCL11, by acting as an outstanding inducer of CXCR3 internalization, reduces the availability of the receptor for the two other IFN-inducible CXCR3 ligands and the platelet-derived CXCR3 agonists CXCL4 and CXCL4L1. Moreover, the authors claimed that CXCL9 and CXCL11 are biased ligands tending toward initiation of β-arrestin recruitment and receptor internalization, respectively. Contrastingly, when discriminating internalization properties of specific CXCR3 splice variants on transfected HEK cells, CXCL11 only modestly provoked internalization of CXCR3A and CXCR3B (74). CXCL11-induced internalization of CXCR3B was independent of β-arrestin signaling (97) CXCL10 induced CXCR3A internalization by 40% within 10 min, while CXCL9-induced CXCR3A internalization manifested three times slower (74). The conflicting results obtained in the two studies regarding the potencies of IFN-inducible CXCR3 ligands to induce CXCR3 internalization may result from the fact that the authors of the first study did not discriminate between CXCR3 splice variants and only took β-arrestin-dependent CXCR3 internalization into account, while others demonstrated that internalization of the receptor may rely also on a β-arrestin-independent machinery (74, 96, 97, 106). Regarding CXCR3B, stimulation with either CXCL9 or CXCL10 induced moderate receptor internalization (74, 97). Remarkably, CXCL9 and CXCL11, but not CXCL10, seem to provoke rapid internalization of CXCR3-alt on transfected cells (74). In summary, the different results from various studies suggest that CXCR3 internalization properties probably depend on the experimental set-up used. Also internalization manifests differently for alternative CXCR3 splice variants. However, one has to keep in mind that the physiological relevance of CXCR3 isoforms *in vivo* remains largely unknown at the moment. Although former studies support the idea that these different receptor variants may fulfill unique roles, transfecting cells with CXCR3 isoforms is artificial and not necessarily recapitulates the *in vivo* situation, implying a need for studies with highly specific antibodies able to discriminate between all three endogenously expressed CXCR3 variants.

### Chemokine-Induced T Cell Polarization through CXCR3

The IFN-inducible CXCR3 interacting chemokines are not only implicated in directed migration of CXCR3 expressing cells, but may also modulate their phenotype (Figure 2). On CD4 positive T cells, stimulation of CXCR3 with CXCL9 or CXCL10 results in downstream phosphorylation of transcription factors “signal transducer and activator” (STAT) 1, STAT4 and STAT5, and subsequent activation of the T-box transcription factor T-bet and the retinoic acid-related orphan receptor γT (RORγT) (77). This suggests that CXCL9 and CXCL10 polarize CD4 positive T cells toward effector cells belonging to the Th1 and Th17 lineages. Contrastingly, CXCL11-induced CXCR3-activation promotes STAT3 and STAT6 phosphorylation and GATA-binding protein 3 (GATA3) activation, thereby driving CD4 positive cells toward the Th2 or Tr1 regulatory phenotype (77). The phenomenon that different ligands may initiate different signaling cascades *via* the same receptor is known as ligand bias. Biased signaling, i.e., the concept that a specific receptor preferentially activates one out of multiple signaling cascades, has become an evidenced phenomenon in the GPCR and chemokine field (13). Moreover, ligand bias, receptor bias and tissue- or cell-specific bias have been described.

### Interaction of IFN-Inducible CXCR3 Ligands with ACKRs and CCRs

In addition to their CXCR3 agonism, at high chemokine concentrations, the IFN-inducible CXCR3 ligands act as full antagonists on CCR3 (Figure 2). They exert this inhibitory effect by competing with the CCR3 ligand CCL11 for receptor binding (108). Evidence exists that CXCL11, which is the most potent CCR3 antagonist, also hinders communication between the chemokines CCL3 and CCL4 and their receptor CCR5 in an antagonistic manner (109). Moreover, CXCL11, is a high-affinity ligand for ACKR1 whereas the two other IFN-inducible CXCR3 ligands bind to ACKR1 only weakly (10, 110, 111). Additionally, CXCL11 but not CXCL9 nor CXCL10 interacts with ACKR3 (10, 112) (Figure 2). In contrast to conventional chemokine receptors, ACKRs have a modified DRYLAIV consensus motif and do not couple to G proteins (10). ACKR1 is a so-called broad spectrum receptor that recognizes CC and CXC chemokines, almost exclusively the ones with an inflammatory nature (17). Experimental evidence suggests that ACKR1 on endothelial cells mainly acts as chemokine transporter and –presenter, thereby shaping the chemotactic gradient. The narrow-spectrum ACKR3 solely interacts with CXCL11 and CXCL12 (17). Activation of ACKR3 does not result in chemotaxis or [Ca^2+^]_i_ mobilization, but offers the cell a survival advantage and impacts cell adhesion and tumor development (112). Indeed, under suboptimal culturing conditions, ACKR3 expression is associated with increased numbers of living cells (112). Moreover, ACKR3 was linked to enhanced expression of vascular adhesion molecules, matrix metalloproteinases (MMPs) and angiogenic factors (113). The receptor mediates antiapoptotic effects, STAT3 signaling and regulates macrophage colony-stimulating factor-induced signaling, thereby promoting tumor development, invasiveness and metastasis (113, 114). Lastly, CXCL10, but not the two other IFN-inducible CXCR3 ligands, has been suggested to display high affinity (*K*_d_ of 1–6 nM) for a functional receptor, different from CXCR3 and GAG, on certain non-hematopoietic cells such as epithelial and endothelial cells (115). This latter CXCL10-specific receptor may be implicated in endothelial cell migration and metastasis.

## GAG-Binding Properties of IFN-Inducible CXCR3 Ligands

A widely accepted concept in the chemokine field is the idea that chemokine-directed leukocyte migration *in vivo* requires interaction between chemokines and GAGs (116–123). GAGs are polysaccharides usually present as part of proteoglycan complexes located in the glycocalyx and extracellular matrix. They are negatively charged and retain chemokines—which are usually highly basic—thereby facilitating generation of a chemotactic concentration gradient that navigates leukocyte migration. GAG-mediated immobilization of chemokines allows chemokine presentation to their receptors on leukocytes. Interaction with GAGs promotes chemokine oligomerization and may also protect chemokines against proteolysis (124–126). All IFN-inducible CXCR3 ligands interact with GAGs and these interactions are essential for their *in vivo* function. For example, recruitment of plasmacytoid DCs requires immobilization of the IFN-inducible CXCR3 ligands on GAGs (127). CXCL9 in particular is an interesting chemokine in the context of GAG interactions due to its exclusive COOH-terminal extension that consists for circa 50% of basic amino acids. Consequently, although CXCL9 is less potent on CXCR3, it is the most efficient GAG-interaction partner of the three IFN-inducible CXCR3 ligands. Our lab previously synthesized several peptides derived from the COOH-terminal domain of CXCL9 and showed that these highly positively charged molecules, specifically a peptide containing the 30 most COOH-terminal residues of full length CXCL9, compete with chemokines for GAG binding, thereby hindering CXCL8- and monosodium urate crystal-induced neutrophil extravasation *in vivo* (47, 128). Their extremely high affinity for GAGs also confers these peptides antiviral properties against GAG-binding viruses such as Dengue virus serotype 2, herpes simplex virus-1 and respiratory syncytial virus (129). Binding of the CXCL9-derived peptides to both soluble and cellular GAGs of different origin was recently evidenced. Shorter peptides (less than 30 amino acids) displayed reduced GAG binding when NH_2_-terminal residues were omitted (128). Moreover, when i.v. injected in mice, the most potent peptide bound to the luminal side of endothelial cells, and prevented adhesion of neutrophils.

Although they lack such a unique positively charged tail as CXCL9, also CXCL10 and CXCL11 interact with GAGs. Moreover, it was demonstrated that *in vivo*, but not *in vitro*, chemotaxis induced by these chemokines, requires GAG interaction (116, 117). Interestingly, residues 20–24, 46, and 47 of CXCL10 were found critical regarding GAG-binding, but are also involved in CXCR3 interaction and signaling (130). Key residues for GAG-binding of CXCL11 are a set of basic amino acids located in the 50s cluster of the chemokine as well as Lys17 (117). However, mutating these residues does not impair its potency on CXCR3. The role of GAGs in regulating the activity of CXCL10 and CXCL11 is probably not limited to their effect on chemokine-induced cell migration. In mice, CXCL10-mediated inhibition of pulmonary fibrosis requires binding of the chemokine to GAGs (131). Furthermore, the antifibrotic properties of CXCL10 after myocardial infarction and inhibition of cardiac fibroblast migration manifest in a CXCR3-independent manner and are probably rooted at the level of CXCL10-GAG interactions (132). Evidence suggests that CXCL10 exerts antiviral properties against Dengue virus by competing for heparan sulfate binding (129, 133). Also its antiproliferative effects on endothelial cells might not require CXCR3-interaction, but may be attributed to GAG-binding (134). Indeed, it was suggested that the inhibitory effects on endothelial cell proliferation and angiostatic properties of CXCL10 are mediated *via* its specific heparan sulfate binding site (135). However, in human melanoma, the angiostatic effects of CXCL10 are mediated through CXCR3, in a GAG-independent manner (136). Furthermore, soluble heparin competes with the three IFN-inducible CXCR3 ligands for binding to endothelial cells, inhibiting transendothelial migration and arterial recruitment of T cells (137). Thus, implying that soluble and immobilized GAGs differently affect chemokine function and suggesting a potential therapeutic anti-inflammatory role for non-anticoagulant heparin derivatives (137). Despite the original vision that the GAG-binding domain is located in the COOH-terminal chemokine region whereas the major domain for receptor interaction is situated rather NH_2_-terminally, it was found that the two major interaction regions are not strictly limited to, respectively, the COOH- and NH_2_-terminus, as mentioned before for CXCL10 (48, 130). This may imply that GAGs and chemokine receptors can show a certain degree of competition for chemokine binding, with a context-dependent outcome. Thus, whether the main interaction partner through which chemokines exert their biological functions is rather a GAG or their cognate protein receptors probably depends on the specific environment.

Also for CXCL11, interference with GAG binding may be interesting from a therapeutic point of view. For example, interfering with CXCL11-GAG interactions using the multifunctional protein TNF-stimulated gene-6, modulates the inflammatory response (138). In mice, CXCL10 shows a higher affinity than CXCL11 for heparan sulfate, which is the most abundant and probably most biologically relevant GAG (*K*_d_ 0.95 ± 0.08 versus 118.3 ± 53.3 nM) (139). However, another study reported that the *K*_d_ of CXCL11 for heparin and heparan sulfate is below 10 nM, and that binding of CXCL11 to these two GAGs is featured by intermediate dissociation and high association, and therefore an overall high affinity, with o-sulfation contributing to the chemokine-GAG interaction (140). The fact that conflicting results are obtained in different studies may suggest that the role of chemokine–GAG binding is context-dependent. Furthermore, regarding studies that rely on surface plasmon resonance technology, the results probably depend on the exact chip coating and GAG modification (e.g., biotinylation), GAG identity (length, amount and density of sulfate and carboxylate groups) and concentrations tested. Also GAG density may codetermine chemokine affinity for GAGs (141). Additionally, the exact affinity observed for a specific GAG is most probably different for a human chemokine and its murine counterpart. To add another level of complexity, it was found that CXCL11 exhibits conformational heterogeneity, and the different states probably display divergent affinities for CXCR3 and GAGs (117). Strikingly, the GAG-binding affinity of CXCL11 in its high GAG-affinity state, equals typical receptor binding affinity. Recently, it was demonstrated that multiple chemokines, including CXCL11, may provoke rearrangement and clustering of GAG chains (141). This phenomenon likely requires chemokine oligomerization, a process that itself is believed to occur in a GAG-dependent way. The exact molecular machinery underlying GAG-mediated chemokine oligomerization remains largely unknown. However, for CXCL10, but also for several other chemokines, GAG-induced oligomerization was found to be required for its *in vivo* activity (116, 119, 142). Oligomeric forms of CXCL9 and CXCL10 likely occur in physiological circumstances (116, 143, 144).

Scientific evidence suggests that GAGs not only modulate the activity of IFN-inducible CXCR3 interacting chemokines directly through ligand binding, but also play a role in upstream chemokine regulation. For example, unfractionated heparin inhibits IFN-γ-induced CXCL9 and CXCL10 production by human breast cancer cells dose-dependently (145). Moreover, unfractionated heparin impacts the IFN-γ response at multiple levels by inhibiting IFN-γ binding to the cells and modulating STAT1 phosphorylation downstream of IFN-γ (145). In contrast to the inhibitory effect of unfractionated heparin on chemokine production, low molecular weight hyaluronan fragments and no other GAGs induce CXCL10 *via* the NF-κB pathway (146). Furthermore, our lab recently found that soluble GAGs interfere with the interaction between CXCR3 and its IFN-inducible ligands (126).

The multidimensional roles of GAGs in chemokine regulation may also be interesting from a therapeutic point of view. A study aiming at improving insights into interactions between chemokines and the extracellular matrix showed that the heparin binding domains of CXCL10 and CXCL11 are also involved in binding of these chemokines to the extracellular matrix proteins fibrinogen and fibronectin (147). Strikingly, this apparently does not apply for CXCL9. Interestingly, CXCL11 synergized with fibronectin in wound healing. These observations underscore the importance of interactions between chemokines and the extracellular matrix components in general, thus suggesting that these are not restricted to GAG binding.

## Regulation of IFN-Inducible CXCR3 Ligand Activity by Posttranslational Modification

**Figure 4:**
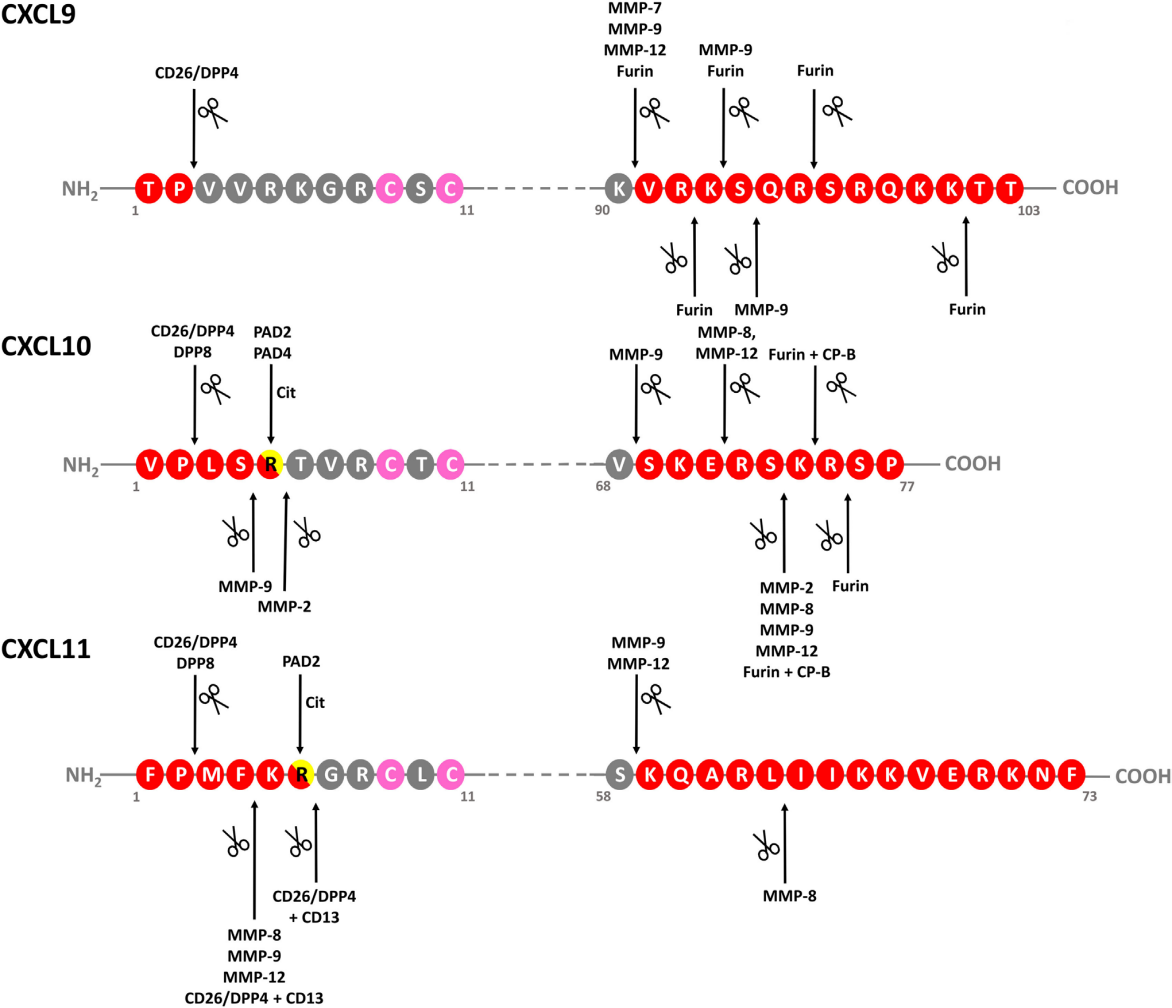
Identified modifications of IFN-inducible CXCR3 ligands. Schematic linear structures of CXCL9, CXCL10, and CXCL11. Enzymes responsible for chemokine modification and corresponding cleavage sites, if determined, are indicated. Conserved Cys residues are indicated in pink. CP-B, carboxypeptidase B; CXCR, CXC chemokine receptor; DPP, dipeptidyl peptidase; IFN, interferon; MMP-, matrix metalloprotease; ND, not determined; PAD, peptidylarginine deiminase.

**Figure 5:**
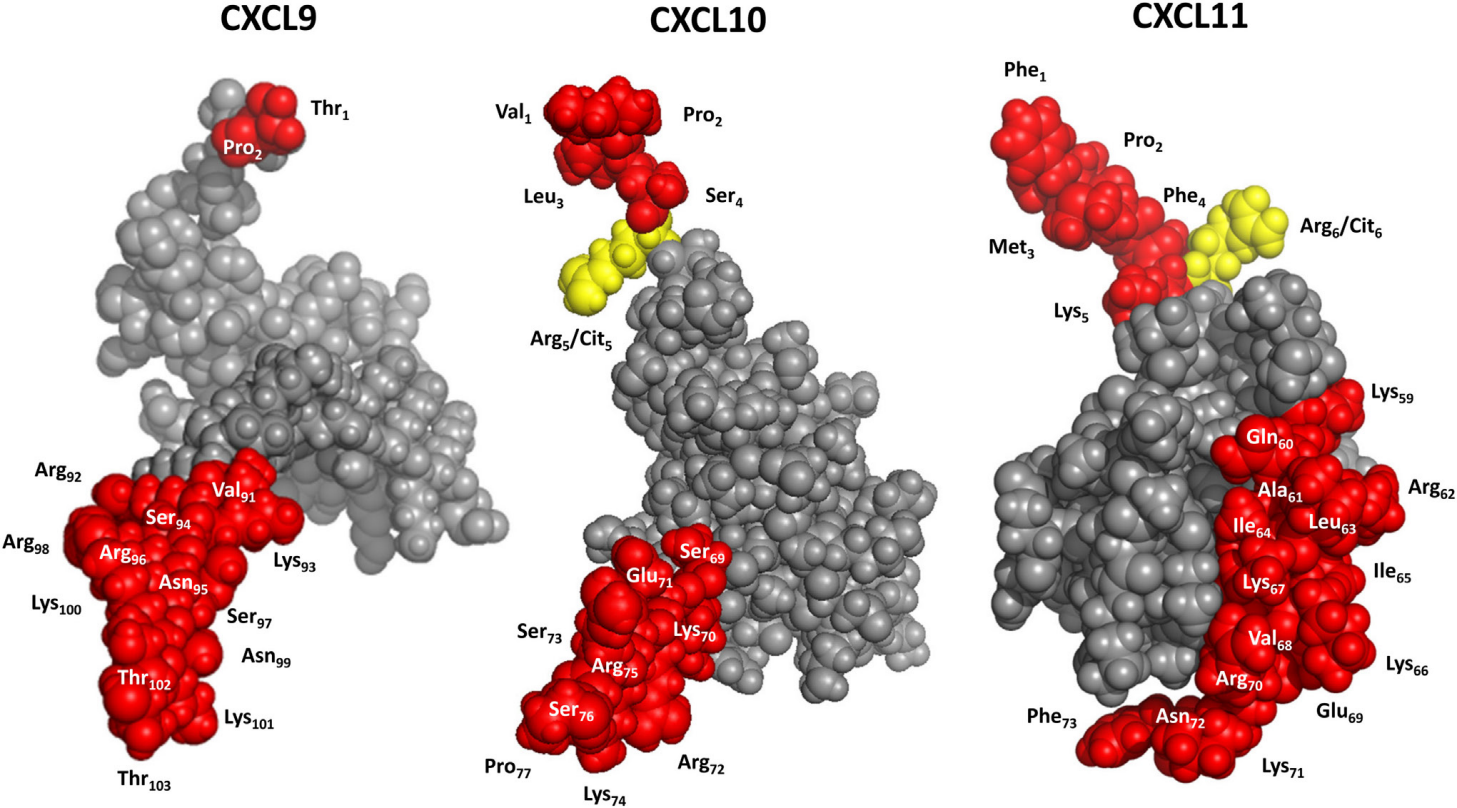
3D models of interferon-inducible CXC chemokine receptor 3 (CXCR3) ligands. CXCL9 was modeled with Swiss-Model software using CXCL8 as a template. CXCL10 and CXCL11 were drafted with PDB accession numbers 1LV9 (84) and 1RJT (85), respectively. Yellow, potential citrullination sites; red, residues that can be cleaved off by enzymatic truncation.

Regulation of the precise chemokine activity and receptor specificity is a multidimensional process with potentially a central role for posttranslational modifications such as proteolytic processing, citrullination, nitration and glycosylation (14–17). Depending on the mode of processing and the chemokine involved, natural modifications may drastically modulate the *in vitro* and *in vivo* chemokine potency. The three IFN-inducible CXCR3 ligands make no exception to this rule (Table 2; Figures 4 and 5). Human CXCL9, CXCL10, and CXCL11 and murine CXCL10 all contain a Pro residue at the penultimate NH_2_-terminal position in their sequence, implying that they are substrates for dipeptidyl peptidase (DPP) 4 or CD26 (46, 148–152). In addition, CXCL10 and CXCL11 are also processed by the related enzyme DPP8 (153). The multifunctional protein CD26 exhibits serine protease catalytic activity and preferably cleaves dipeptides from substrates with a (hydroxy)Pro or Ala in the second position at the NH_2_-terminus. The IFN-inducible CXCR3 ligands, especially human CXCL10 and CXCL11 and murine CXCL10, are short half-life CD26 substrates (148, 149). For all human IFN-inducible CXCR3 interacting chemokines, CD26-mediated processing results in loss of chemotactic activity with retention of angiostatic features. Moreover, this site-specific truncation converts CXCL10 and CXCL11 into CXCR3 antagonists. Natural CD26-processed isoforms of CXCL10 and CXCL11, i.e., CXCL10(3–77) and CXCL11(3–73), were isolated from cell culture supernatant, while CXCL10(3–77) was also detected in murine and human body fluids (46, 150, 151, 153–156). Coexpression of CXCL10 and membrane-bound CD26 was found on stimulated fibroblasts, suggesting the existence of a negative feedback machinery controlling CXCL10-dependent chemotaxis (46). Indeed, regarding the effect of CD26-mediated truncation for the biological activity of IFN-inducible CXCR3 ligands, one could speculate that CD26 expression and/or specific enzymatic activity may be reduced when the role of these chemokines becomes particularly relevant. However, this hypothesis has not been proven yet (157). CXCL10 was claimed to be the most relevant chemokine biomarker in patients with rheumatoid arthritis, but also large numbers of CD26-expressing cells were found in these patients (158–160). Remarkably, although rheumatoid arthritis was not associated with altered levels of soluble CD26, the specific activity of the enzyme was reduced, probably because of glycosylation (161, 162). In mice, inhibition of CD26 was found to protect CXCL10 from inactivation, resulting in enhanced T cell infiltration into the tumor tissue, thereby improving not only the natural antitumor immunity but also the response to existing immunotherapies (156). In humans, CD26-mediated cleavage of CXCL10 correlates with failure to spontaneously clear viral hepatitis C infection (163, 164). Recently, direct evidence in favor of CD26 inhibition to preserve full length, active CXCL10 and prevent cleavage into the inactive isoform CXCL10(3–77), was found in humans (150). Interestingly, we recently demonstrated that GAGs protect human CXCL9, CXCL10, and CXCL11 against processing by CD26 in a dose-dependent manner (126).

**Table 2:** Posttranslational modifications of IFN-inducible CXCR3 ligands.

CXCR3 ligand	Mode of processing	Responsible enzyme(s)	Confirmation of processing	Biological consequences	Natural source of modified chemokine	Reference
CXCL9	NH_2_-terminal cleavage	CD26/DPP4	*In vitro*	Loss of signaling and chemotaxis on leukocytes	ND	(148, 149)
				Retains antiangiogenic activity		

	COOH-terminal cleavage	Furin	*In vitro*	ND	ND	(165)
		MMP-7, -9, -12	*In vitro*	ND	ND	(166, 167)
		ND	*In vitro*	Reduced calcium response	THP-1 cells, human peripheral blood monocytes	(168)
		ND	*In vitro*	ND	PBMCs	(47)

	Degradation	MMP-8	*In vitro*	Inactivation	ND	(166)

CXCL10	NH_2_-terminal cleavage	CD26/DPP4, DPP8	*In vitro, in vivo* (CD26/DPP4)	Inactivation, CXCR3 antagonist	Fibroblasts, osteosarcoma cells, human and murine plasma	(46, 148–150, 153–156)

	COOH-terminal cleavage	Furin + CP-B	*in vitro, in vivo*	Unaltered *in vitro* activity	IFN-γ-stimulated primary human keratinocytes	(165)
		MMP-8, -12	*In vitro*	ND	ND	(166, 169)

	Cleavage at both termini	MMP-2, -9	*In vitro*	ND	ND	(166, 167)

	Degradation	MMP-7, -9	*In vitro*	Inactivation	ND	(166, 169)

	Citrullination	PAD2, PAD4	*In vitro*	Reduced activity	dsRNA- and IFN-γ-stimulated PBMCs	(48)

CXCL11	NH_2_-terminal cleavage	CD26/DPP4, DPP8	*In vitro*	Inactivation, CXCR3 antagonist	IFN-γ-stimulated keratinocytes	(148, 149, 151, 153)
		CD26/DPP4 + CD13	*In vitro*	Reduced angiostatic activity	dsRNA- and IFN-γ-stimulated PBMCs and fibroblasts	(170)

	Cleavage at both termini	MMP-8, -9, -12	*In vitro*	NH^_2_^-terminal cleavage: CXCR3 antagonist increased GAG binding. These effects are lost upon subsequent COOH-terminal processing	ND	(169)

	Degradation	MMP-7, -12	*In vitro*	Inactivation	ND	(169)

	Citrullination	PAD2	*In vitro*	Reduced activity	ND	(48)

*CP-B, carboxypeptidase B; DPP, dipeptidyl peptidase; IFN, interferon; MMP-, matrix metalloprotease; ND, not determined; PAD, peptidylarginine deiminase*.

In addition to NH_2_-terminal truncation, also the COOH-terminal domain of IFN-inducible CXCR3 ligands can be naturally cleaved (Figures 4 and 5). COOH-terminal processing seems biologically most relevant for CXCL9. As early as in 1995, it was reported that natural CXCL9 displays a high degree of COOH-terminal heterogeneity, with most of the secreted chemokine lacking up to 25 amino acids (168). In our lab, COOH-terminally shortened isoforms and no full length CXCL9 of 103 amino acids was detected in cell culture supernatant from PBMCs stimulated with dsRNA and IFN-γ, and from fibroblasts stimulated with dsRNA, IFN-γ, or LPS (47). Enzymes responsible for COOH-terminal processing of CXCL9 *in vitro* are furin and MMP-7, MMP-9, and MMP-12 (165, 166, 169). However, these are extracellular proteases, while it has been suggested that natural COOH-terminally truncated CXCL9 isoforms may result from proteolysis even before chemokine secretion, at least when considering CXCL9 produced by the Chinese Hamster Ovary cell line (168). In addition to the possibility of yet uncharacterized key player enzymes and processing mechanisms, also the exact biological impact of COOH-terminal cleavage on CXCL9 activity *in vivo* remains largely unknown. However, the truncated isoforms are less potent in *in vitro* calcium mobilization assays, underscoring the idea that the physiological function of the COOH-terminal chemokine region is not limited to merely GAG binding (168). Worth mentioning, COOH-terminally truncated CXCL9 species do not antagonize the activity of the native chemokine (168).

*In vitro*, the COOH-terminal domain of CXCL10 can be cleaved by MMP-8 and MMP-12, while the related enzymes MMP-2, MMP-7 and MMP-9 rather process the chemokine at both termini or completely destroy the protein (166, 167, 169). For CXCL10, COOH-terminal processing by furin plus carboxypeptidase B (CP-B) was confirmed *in vivo* (165). However, no evidence was found for altered biological functioning of CXCL10 modified by furin/CP-B. Conflicting results have been reported regarding cleavage of CXCL9 and CXCL10 by MMP-8 and MMP-9. Two studies reported MMP-9-mediated processing of CXCL10, with one also demonstrating cleavage of the chemokine by MMP-8 (166, 167). The authors of the latter study also reported processing of CXCL9 by both enzymes (166). In contrast, in a third study, cleavage of CXCL9 and CXCL10 by the two MMPs could not be confirmed (169). In the context of proteolytic processing by MMPs, CXCL11 is a highly remarkable ligand. On the one hand, removal of its four most NH_2_-terminal residues by MMP-8, MMP-9, or MMP-12 turns the chemokine into a CXCR3 antagonist with increased heparin binding properties (169). However, on the other hand, upon subsequent cleavage by these MMPs near position 58 in the COOH-terminal domain of CXCL11, the antagonistic features and altered GAG binding of CXCL11 that resulted from NH_2_-terminal processing are lost.

Thus, all human IFN-inducible CXCR3 ligands are substrates for specific MMPs and CD26, with CXCL11 being most susceptible to cleavage by most of these enzymes, at least *in vitro* (148, 149, 169). Although processing of CXCL10 has been studied more intensively in an *in vivo* context, *in vivo* evidence of natural modification of CXCL11 is scarce at the moment. The reason why CXCL11 has been investigated to a lesser extent *in vivo* may be rooted at multiple levels. First, concentrations of the naturally secreted chemokine are often rather low compared to CXCL9 and CXCL10, at least when considering cell culture supernatant from stimulated endothelial cells (107) or leukocytes (35). Additionally, several independent research groups suggest that C57/BL6 mice, one of the most routinely used animal models, do not express endogenous CXCL11 (171, 172). Lastly, regarding CD26-mediated processing specifically, murine CXCL11 and CXCL9 do not contain a Pro in the penultimate NH_2_-terminal position, implying that they are no substrates for CD26. However, the short *in vitro* half-life of human CXCL11 in the presence of various proteolytic enzymes, combined with the drastic effects of modification on its biological activity, imply that proteolytic modification of CXCL11 in humans may be highly relevant.

In addition to enzymatic modification generating truncated isoforms of the IFN-inducible CXCR3 ligands, also site-specific citrullination by peptidylarginine deiminases (PADs) has been evidenced for CXCL10 and CXCL11 (Figures 4 and 5). Specifically, PADs can deiminate the positively charged Arg residue toward the neutral amino acid citrulline (Cit) at the NH_2_-terminal positions 5 and 6 of CXCL10 and CXCL11, respectively (48). Compared to native CXCL10 and CXCL11, [Cit^5^]CXCL10, and [Cit^6^]CXCL11 show impaired T cell chemotactic activity and [Ca^2+^]_i_ mobilization, and reduced GAG-binding, while their receptor binding properties remain unaffected. Moreover, natural [Cit^5^]CXCL10 was found in conditioned medium from stimulated PBMCs. Thus, despite the fact that citrullination increases the molecular mass of the substrate involved by only 1 mass unit, significant consequences for the biological chemokine function may result. Furthermore, emerging evidence suggests that protein citrullination becomes more important in an inflamed environment (173, 174).

Although the biological effects of multiple modifications of IFN-inducible CXCR3 ligands has been examined *in vitro* or even *in vivo*, the exact underlying molecular pathways remain largely unknown. For example, it is yet to be elucidated whether specific modifications selectively affect Gα protein- or β-arrestins-dependent pathways. Moreover, one could speculate that posttranslational processing may convert the chemokine into a biased ligand that preferentially induces activation of a specific signaling cascade through its receptor. Furthermore, the fact that most enzymes known to process IFN-inducible CXCR3 agonists are upregulated in an inflamed environment, suggests that chemokine modification may become more relevant during pathophysiological circumstances. Consequently, one could speculate that modified chemokine isoforms can be biomarkers for specific diseases. This hypothesis is favored by the observation that CD26-truncated CXCL10 correlates with HCV disease activity (149, 164).

## Unprecedented Versatility of IFN-Inducible CXCR3 Ligands as Exemplified in Angiogenesis, Cancer and Inflammation

### Relevance of IFN-Inducible CXCR3 Ligands in Disease and Therapy

The inflammatory nature and chemotactic activity for T cells and NK cells, among others, implies that CXCL9, CXCL10, and CXCL11 may be key players in inflammation and autoimmunity. In addition, their antiangiogenic effect, which was first confirmed *in vitro* and *in vivo* for CXCL10 (175, 176), implies that the biological function of IFN-inducible CXCR3 ligands extends beyond their hallmark function of directing migration of CXCR3 expressing leukocytes. Meanwhile, also the angiostatic properties of the two other IFN-inducible CXCR3 agonists and of the platelet-derived CXCL4 and CXCL4L1 were demonstrated (78, 82, 149, 177–179). Addition of CXCR3 neutralizing antibodies was found to abrogate migration of human endothelial cells and to inhibit the CXCL4L1-induced antiangiogenic activity in the rat cornea (78, 180). Moreover, anti CXCR3 antibodies prevented inhibition of tumor growth and CXCL4L1 had no effect on tumor growth in CXCR3^−/−^ mice (78). These results may indicate that the angiostatic effect of the CXCR3 ligands is a CXCR3-dependent phenomenon, at least when chemokines are exogenously added. Moreover, in a murine model of *Candida albicans* uveitis, neutralization of CXCR3 increased angiogenesis in the cornea, indicating that murine CXCR3 in this model provides a negative feedback on vessel formation (181). A similar observation was made *in vitro* in cocultures of human pericytes and endothelial cells. The pericytes suppress endothelial network formation, but this inhibitory action was reversed by adding neutralizing anti-CXCR3 antibodies (182). Also in a murine model of liver fibrosis, in which angiogenesis and fibrosis are induced by carbon tetrachloride (CCl_4_), CXCR3 has been shown to dampen angiogenesis (183). Indeed, angiogenesis was attenuated in CXCR3^−/−^ mice, compared to wildtype mice. Enhanced neoangiogenesis and VEGF/VEGFR2 expression in *Cxcr3*^−/−^ mice compared with wildtype littermates was strongly linked to fibrosis progression. There are also indications that CXCR3A is not the receptor mediating the antiangiogenic effects of its ligands. Administration of human CD26-truncated forms of both CXCL9 and CXCL10, which do not induce a calcium signal or chemotaxis through CXCR3A, retain antiangiogenic properties in the rabbit cornea assay (149). This implies that either alternative signaling pathways through CXCR3, receptor variants or alternative receptors, with a potential role for GAGs, are involved in the antiangiogenic activity. In addition, although an inhibitory effect of CXCR3 ligands was observed in many experimental models, to our notice there is no direct evidence showing the role of endogenously expressed CXCL9, CXCL10, and CXCL11 in angiogenesis in humans.

The fact that they are believed to regulate angiogenesis self-evidently suggests that these chemokines may also be involved in tumor biology and hematological malignancies (24). In general, differential and context-dependent roles have been attributed to IFN-inducible CXCR3 ligands in numerous *in vivo* disease models (24, 25). However, summarizing all roles that were claimed for IFN-inducible CXCR3 ligands in specific diseases of inflammatory and non-inflammatory origin, and the potential therapeutic relevance of interfering with the IFN-related CXCR3 chemokine network was previously done and extends beyond the scope of this review (24, 184–194). Hence, within the present review we included selected examples of IFN-inducible CXCR3 ligands in a biological context, and aim to illustrate that the multidimensional regulatory machinery and ligand-specific properties as indicated above may offer IFN-inducible CXCR3 ligands their non-redundant and context-dependent activities. Thus, we speculate that ligand exclusivity is the biological consequence of unprecedented versatility delineating the IFN-related CXCR3 chemokine system (Figure 2).

### Examples of Non-Redundancy and Dominance of Specific IFN-Inducible CXCR3 Ligands

First of all, regarding the *in vivo* chemotactic and angiostatic activity of individual IFN-inducible CXCR3 ligands, one has to keep in mind that most experimental models do not take into account the exclusive temporal and spatial expression patterns that seem inherent to individual ligands. For example, an early model claiming that IFN-inducible CXCR3 ligands recruit T cells to lung tissue with similar efficacy did not consider this aspect of chemokine regulation (195). The fact that CXCL9, CXCL10, and CXCL11 may be secreted at specific time points by specific cells in response to specific stimuli forms a first line of complexity when aiming to unravel their individual contributions during immune events. Although the vast majority of published data considering IFN-inducible CXCR3 ligands points toward non-redundant roles for individual ligands *in vivo*, few studies evidenced that ligand redundancy may exist in particular milieus. For example, loss of either CXCL9 or CXCL10 was countervailed for by the presence of the other in a murine model of obliterative bronchiolitis (196). Nevertheless, multiple reports exist on key roles for individual IFN-inducible CXCR3 ligands in particular diseases, emphasizing that ligand dominance may occur more commonly between IFN-inducible CXCR3 ligands. For example, neither CXCL9 nor CXCL11 could compensate for the loss of CXCL10 activity in experimental models for infection with Dengue virus and West Nile virus (197, 198). In addition to a dominant role for CXCL10 in these viral infection models, the chemokine was demonstrated to also fulfill non-redundant activities in a mouse model of vitiligo (199). In pulmonary sarcoidosis, CXCL10, and not CXCL9 nor CXCL11, is released by bronchoalveolar lavage cells (200). One has to keep in mind that most research conducted on the IFN-related CXCR3 chemokine system involves CXCL10, although this does not mean that CXCL10 is always the most relevant IFN-inducible CXCR3 ligand. In line with this idea, an essential role for CXCL9 but not CXCL10 was demonstrated for example during murine kidney inflammation (201). CXCL9 also was specifically associated with macrophage activation syndrome complicating systemic juvenile idiopathic arthritis (202). The currently available studies on *in vivo* functions of CXCL11 are limited, entailing a potential risk for underappreciating this chemokine. To support the idea that also CXCL11 may be a most relevant member of the IFN-inducible CXCR3 ligand family, a recent study suggested potential association between a homozygous CXCL11 variant with an increased risk of contact allergy (203). In addition, and inherent to the lack of CXCR3 spliced variants in mice, most animal models do not allow to investigate a potential role for a cell-specific expression of receptor variants and different signaling pathways through these variants in patients.

### Examples of Collaboration and Mutual Antagonism of IFN-Inducible CXCR3 Ligands

In addition to ligand dominance, also the simultaneous expression of more than one IFN-inducible CXCR3 ligand can be prototypical for a disease, and eventually a certain degree of collaboration may exist between the three ligands. For example, upregulation of CXCL10 and CXCL11 was found in patients with *Chlamydia Trachomatis* (204). Production of all three IFN-induced CXCR3 ligands was enhanced in cerebral malaria in mice, and mouse strains that were more susceptible to the disease had an enhanced expression of the CXCR3 receptor (205). Moreover, both CXCL9 and CXCL10 were reported to be required for development of cerebral malaria in mice (206). Also the host’s ability to control herpes simplex type 2 infection was found to involve CXCL9 and CXCL10 (207). Regarding diabetes, production of all three IFN-inducible CXCR3 ligands by pancreatic β-cells may precede disease onset (208). Nevertheless, data from a study on rejection of allograft heart transplants provided an example of antagonism between IFN-inducible CXCR3 ligands, since increased levels of CXCL9 suppressed CXCL10 expression in this context (209). Interestingly, in addition to a potential mutual effect on each other’s expression or function, also crosstalk between IFN-inducible CXCR3 ligands and non-CXCR3 interacting chemokines may occur. Specifically, CXCL9, CXCL10, and CXCL11 increased the sensitivity of plasmacytoid DCs to the constitutively expressed CXCR4 ligand CXCL12 by 20–50-fold (210). Moreover, collaboration seems not only restricted to chemokines, since synergy was also described between CXCR3 and the T cell receptor (211). In contrast, coexpression of the atypical receptor ACKR4 completely inhibited CXCR3-mediated chemotaxis (212).

Due to their potential to inhibit angiogenesis and recruit antitumor leukocytes, IFN-inducible CXCR3 ligands may function as tumor suppressors (213). For example, in colorectal cancer high levels of CXCL9 and CXCL10 correlated with increased disease-free survival (214). It was suggested that increasing CXCL9 and CXCL10 might be an effective immunotherapeutic approach in this type of disease (215). Moreover, in melanoma enhanced CXCL9 and CXCL10 correlated with reduced metastasis (216). As a last example of IFN-inducible CXCR3 interacting chemokines as potential tumor suppressors, CXCL9 and CXCL10 were found to promote the natural antitumor immunity of the host also in gastric cancer (217, 218). Nevertheless, the capacity to recruit immune cells such as regulatory T cells (219, 220) also implies that CXCL9, CXCL10, and CXCL11 can shape the microenvironment toward a rather tumor-promoting milieu. Indeed, a critical role for CXCL10 and to a lesser extent CXCL9 and CXCL11 was found in promoting breast cancer development (221). CXCL10 and CXCL11 were also associated with poor prognosis in a model for colorectal cancer by initiating macrophage infiltration (222).

### Final Remarks

Most reports on IFN-inducible CXCR3 ligand activities do not consider the facts that three CXCR3 isoforms have been identified, that also two platelet-derived chemokines interact with CXCR3 and that CXCL11, but not CXCL9 or CXCL10, is a ligand for ACKR3. This consequently adds an important dimension of complexity to the IFN-related CXCR3 chemokine system. Indeed, the fact that eventually opposing downstream pathways may be initiated by different CXCR3 isoforms may explain the differential activity of a specific IFN-inducible CXCR3 ligand (74). In addition to multiple CXCR3 isoforms, also several isoforms of IFN-inducible CXCR3 ligands themselves may exist *in vivo* (14–17). As mentioned, numerous studies reported association between up- or downregulation of one or more IFN-inducible CXCR3 ligands and a specific disease state. However, it is usually unclear whether the authentic, full length chemokine or a modified isoform with a drastically different biological activity is most abundant. Indeed, the slowly appearing scientific evidence that *in vivo* functioning of IFN-inducible CXCR3 ligands is modulated by posttranslational modification may also explain apparently opposing activities of IFN-inducible CXCR3 ligands. This idea is supported by the fact that enzymes known to process these chemokines into molecules with altered biological activity are naturally upregulated in specific diseases. Overall, the versatility that seems inherent to the IFN-related CXCR3 chemokine system emphasizes the need for antibodies and sensitive techniques able to discriminate between specific forms of the IFN-inducible CXCR3 variants in biological samples and *in vivo*. We speculate that improved insights into the presence and abundance of both receptor and chemokine isoforms would dramatically contribute to our understanding of the IFN-related CXCR3 chemokine network. Moreover, due to their apparent roles in infection, inflammation, angiogenesis, and cancer, thoroughly understanding the IFN-related CXCR3 chemokine system would be of clinical value, both from a diagnostic and therapeutic point of view.

## Author Contributions

MM wrote the initial version of the manuscript with assistance of all other authors. The manuscript was approved by all authors.

## Conflict of Interest Statement

The authors declare that the research was conducted in the absence of any commercial or financial relationships that could be construed as a potential conflict of interest.

## Abbreviations

ACKR, atypical chemokine receptor; cAMP, cyclic adenosine monophosphate; CP-, carboxy peptidase; DC, dendritic cell; DPP, dipeptidyl peptidase; GATA3, GATA-binding protein 3; GPCR, G protein-coupled receptor; HUMECs, human microvascular endothelial cells; IFN, interferon; IP-9, IFN-γ-inducible protein-9; IP-10, IFN-γ-inducible protein of 10 kDa; IRSE, interferon response element; I-TAC, IFN-inducible T cell α chemoattractant; IL-, interleukin; NK, natural killer; LPS, lipopolysaccharide; Mig, monokine induced by IFN-γ; MMP, matrix metalloprotease; NF-κB, nuclear factor kappa B; PAD, peptidylarginine deiminase; PBMCs, peripheral blood mononuclear cells; PHA, phytohemagglutinin; RORγT, retinoic acid-related orphan receptor γT; STAT, signal transducer and activator; TNF, tumor necrosis factor; γIRE, γ-interferon response element.
